# Challenges faced by edentulous patients (EDPs) during complete denture prostheses (CDP) service delivery in Fiji – a qualitative study

**DOI:** 10.1186/s12913-022-08136-6

**Published:** 2022-06-04

**Authors:** Meenal Nand, Masoud Mohammadnezhad

**Affiliations:** 1 School of Dentistry and Oral Health, Fiji National University, Suva, Fiji; 2 School of Public Health and Primary Care, Fiji National University (FNU), Suva, Fiji

**Keywords:** Challenges, Edentulous patients, Complete dentures prostheses, Service delivery, Fiji

## Abstract

**Background:**

Edentulism is a state of having no teeth in the oral cavity, an irreversible condition popularly known as the marker of disease burden on the oral health. For edentulous patients (EDPs) Complete Denture Prostheses (CDP) is a means to improve oral health. Due to limited studies in this area, this research aimed to explore the challenges faced by the EDPs during CDP service delivery in Fiji.

**Methods:**

A directed descriptive qualitative study was conducted among EDPs attending Dental Prosthetic Clinics (DPCs) at the four centres in Fiji under purposive sampling. A semi-structured questionnaire with open ended questions was used for in-depth interview with EDPs who had worn CDP for 1 year via telephone. Participant responses were recorded, transcribed verbatim and analyzed manually using thematic analysis.

**Results:**

A total of 30 EDPs participated in the study. Six themes were identified as challenges faced by the EDPs during CDP service delivery in Fiji: lack of information, non-compliance, overwhelmed by illness, reduction on quality of life (QoL) financial barriers to CDP treatment care and personal acceptance. 77% of EDPs felt they did not receive adequate information on CDP when attending DPCs which indicated the non-compliance nature irrespective of using and cleaning CDP on a daily basis. The Dental Professionals (DPs) at the DPCs gave their full support in attending to the CDP challenges faced by the EDPs in Fiji during the review clinics.

**Conclusion:**

The EDPs expressed a variety of challenges that was faced while seeking appropriate CDP services in Fiji. The findings demonstrate the need to explore approaches to develop patients’ engagement practices during CDP service delivery by the Dental professionals (DPs), which leads to effective oral health promotion in Fiji for CDP wearers.

## Background

Complete edentulism is an irreversible condition which is explained as the known marker of disease burden for oral health where an oral cavity is without any teeth [1–3]. Patients who suffer from this state of complete edentulism undergo numerous health conditions such as loss of speech, in appropriate esthetics and poor mastication which affects the Quality of Life (QoL) of Edentulous Patients (EDPs) [3–6]. At a global stage, the World Health Organization (WHO) databanks indicated that the incidence of complete edentulism has been estimated between 7 and 69% respectively [7–9]. The oral health services in Fiji are responsible for the delivery of sustainable oral health programs for all citizens in the country through its highly comprehensive legislative, promotional, curative, preventive and rehabilitative approaches to encourage retention of teeth in leading towards better QoL [10].

Primarily, complete dentures are provided to the edentulous population to assist them in the immediate mastication processes as this therapy brings about improved oral health outcomes in relation to its aesthetics and maintenance requirements [3, 11–13]. Majority of Complete Denture Prostheses (CDP) service is being utilized by the elderly population in Fiji [14–16]. In the process of receiving new dentures, patients tend to forget that there will be challenges expected to be encountered when receiving new CDP. While the incidence of complete edentulism [17] in Fiji tends to increase due to rise in Non-Communicable Diseases (NCDs), of which oral health is also a component, the amount of extraction rate will be constant for some period of time leading to challenges the EDPs will face when seeking appropriate CDP services.

There has been no previous study done on CDP where patients’ views on the challenges they faced was studied. There are a few previous studies on complete denture wearers [18–20] had been conducted where the complaints related to CDP which included both upper and lower dentures were considered. This limited information available for a developing country like Fiji makes room for further research to be conducted in this area which plays a vital role in the lives of the EDPs in terms of exploring the challenges they had faced while receiving CDP services in Fiji.

To fill the knowledge gap that was identified and ignored in the society due to the state of edentulism, it was highly relevant to have open discussions with EDPs on the challenges they faced while receiving CDP services in Fiji. This study aimed to explore the challenges of EDPs while receiving CDP related services in Fiji.

## Methodology

### Design and setting

This was a directed qualitative study which used semi-structured interviews to conduct research. The Consolidated Criteria for Reporting Qualitative Research (COREQ) was applied to this research to ensure study rigor [21].

This country wide study was conducted at the four dental prosthetic clinics in Fiji via telephone from 1st August 2021 to 1st September 2021. This was the best way to conduct research amidst the Coronavirus Disease 2019 (COVID-19) pandemic in exploring challenges of participants in a qualitative study. This study was conducted at the Fiji National University (FNU) Dental clinic and the three Divisional Dental Prosthetic Clinics (DPCs) in Fiji namely: Colonial War Memorial Hospital (CWMH), Lautoka Hospital and Labasa Hospital DPCs. The respective clinics were selected for the study because these were the only specialty centres in Fiji providing CDP services for the study samples.

### Participants

Through communications with Head of School (HOS) of School of Dentistry and Oral Health (SDOH) and the Principal Dental Officers (PDOs) of the 3 respective government DPCs, purposive sampling was employed to recruit participants who differed in characteristics. The inclusion criteria for EDPs were as follows: CDP wearers, aged 18 years and over, male and females, self –identified Fijians attending the four selected clinics in the study period, patients well oriented to time and place following the complete denture post-operative advice promptly. EDPs with partial denture, suffering from Temporomandibular Joint (TMJ) disorders, psychological defect, extremely resorbed ridge based on clinical assessment and those who did not wish to participate were excluded from the study.

A sample size of 30 participants was recruited for the study which included 8 participants from FNU dental clinic, 8 participants from CWM Hospital, 7 participants from Lautoka Hospital and 7 participants from the Labasa Hospital. The interview was conducted over telephone with the 30 participants until data saturation was achieved.

### Data collection

Data was collected via semi-structured questionnaire with open ended questions. The original interview guide was developed (Table 1) based on a literature review and the research questions of the study. There were 2 sections of the questionnaire with a total of 10 questions of which section 1 comprised of the demographic information of EDPs like age, gender, ethnicity, marital status, employment status and division with 6 questions while section 2 had 4 open ended questions associated with the challenges faced by EDPs during CDP service delivery in Fiji translated in English, Hindi and I-Taukei languages. Considering the participants’ health status and availability, the in-depth interview was conducted based on participant’s preferences to know more depth and their respective experiences.

**Table 1:** In-depth interview guide

**Section 1**	
Patient Name:	
Age:	
Gender	
Ethnicity:	Division:
Marital Status:	Employment:
**Section 2: Patient Challenges**	
1. What are your perceptions on the challenges faced regarding your CDP in Fiji?	
2. How are these challenges interfering with the daily wearing of your CDP?	
3. What strategies are you adopting in order to ensure you wear your complete dentures?	
4. Are these strategies helping you in overcoming the challenges you face while wearing your complete dentures?	

Contact details of potential EDPs were provided by the 4 dental prosthetic centres in Fiji who were then contacted via telephone call to request for their participation 1 week before the commencement of the study. Once the EDPs were fully aware of the process that was informed to them from the information sheet, then they gave their approval for participation by verbally agreeing to participate after the consent form was fully read to them over the telephone prior to the commencement of the interview. The main researcher declared that there are no conflicts of interest in this work thus welcomed comprehensive discussions of every aspect of the patients’ care related to CDP service delivery in Fiji. At the beginning of the IDI, each participant answered the sociodemographic sheet prior to commencement of the interview. All the interviews were digitally recorded (range: 30–35 minutes). Memos were written down, including the pauses, the expressions of emotions such as “uh” and “ummm” [22]. Sampling was continued until no new information emerged and data saturation was achieved [23].

### Data analysis

Data analysis started as soon as each interview was completed. All the contents of the interview were transcribed to a word file. Transcription was done Verbatim by the principal researcher on the same day of in-depth interview [22]. All forms of identifiers from the data was removed upon repeated reviewing of the transcribed data. The data was de-identified during the transcription and presentation of quotes was anonymized. Manual thematic analysis was undertaken for encoding qualitative information and identifying themes, subthemes and codes found in information that at minimum described and organized the possible observations and at maximum interpreted aspects of the phenomenon [24] which was then cross-checked by the second researcher. The five steps involved in the analysis process were read and re-read of transcriptions to get general views, code quotes in text, group commonly coded quotes into categories, collapse similar categories into themes/sub-themes and select quotations to illustrate themes and sub-themes [24].

### Study rigor

The criteria’s that met the trustworthiness of the study which were transferability, credibility, confirmability and dependability [25]. Strategies undertaken to ensure transferability in this study was by conducting IDI until data saturation was reached and the use of purposive sampling in this study. Strategies adopted to make sure of credibility was by digitally recording the interviews and the transcription was performed same day by the main researcher. In addition, the participants were contacted and advised on the study before they gave their approval to be interviewed together with thorough discussion was done with supervisor at every stage and chapter of the study. For confirmability, strategies taken was the conceptual triangulation which was adopted in exploring qualitative data through utilization of frameworks. For dependability, the IDI questions were same for all the participating EDPs, initiation and execution of study was performed through thorough systematic research of the existing literatures related to the study. Furthermore, all the data was coded and checked by the supervisor and the transcriptions were re-read and noted to correct any possible errors.

### Ethical consideration

The College Health Research Ethics Committee (CHREC) of Fiji National University (FNU) approved the study (reference: 033.21). All participating EDPs were fully informed about the content of study. The voluntary and confidential nature of the study was highlighted and thoroughly explained to the EDPs via telephone call where these the participants had the right to withdraw from study at any time. In addition, the fact that refusal or withdrawal from the study would not affect patient care as this was a low risk study. Verbal informed consent were obtained via telephone prior to the commencement of the interviews.

## Results

Thirty EDPs had participated in the interview as revealed in Table 2 below highlighting the demographical characteristics of EDPs which included gender, age groups, ethnicity, marital status, employment status and the division the participant was from. Majority of participants were female (60%) as compared to male participants (40%). In terms of age group, majority of the participants were from 60 to 70 yrs. of age (60%). For ethnicity, majority of the participants interviewed were Fijian of Indian Decent (FID) (87%) while the remaining percentage constituted to the I-Taukei (IT) (13%). Looking at the marital status for the EDPs, majority of the participants were married (97%) while remaining were divorced (3%). In addition, based on employment status of participants, majority participants were unemployed (93%) while rest of them were employed (7%). Furthermore, on the divisions, most participants were from central division as it consisted of the CWMH dental clinic and the FNU dental clinic constituting to majority participants (54%) while northern division and western division constituted to same number of participating EDPs (23%) respectively.

**Table 2:** Demographical characteristics of participating EDPs for In-depth interview (*n =* 30)

Characteristics		Frequency (n)	Percentage (%)
Gender	Male	12	40
	Female	18	60
Age groups	50-60 yrs	7	23
	60-70 yrs	18	60
	70-80 yrs	5	17
Ethnicity	I-Taukei	4	13
	Fijian of Indian decent	26	87
Marital status	Single	0	0
	Married	29	97
	Divorced	1	3
Employment status	Employed	2	7
	Domestic duties	28	93
Division	Northern	7	23
	Western	7	23
	Central	16	54

**Fig. 1:**
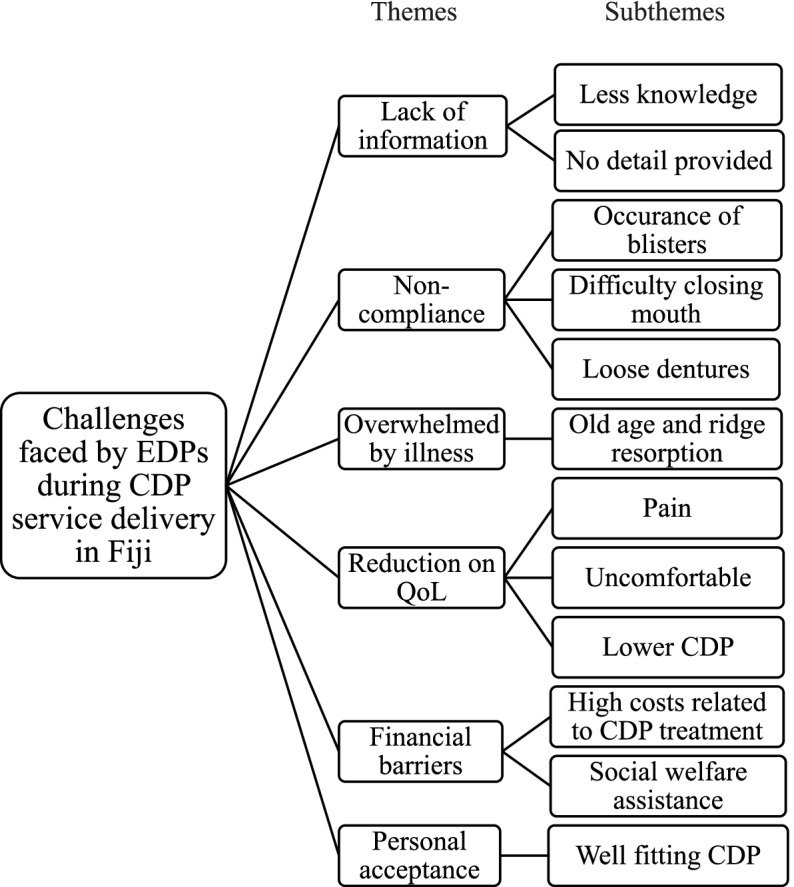
Themes and subthemes from IDI analysis

Six themes were identified from the data analysis as illustrated in Fig. 1 which included lack of information, non-compliance, overwhelmed by illness, reduction on QoL financial barriers to CDP treatment care and personal acceptance by the EDPs. Several subthemes were also discovered and illustrated with quotes. Each EDP was given a participant number like P1, P2 …

### Theme 1: lack of information

This theme reflected the information the EDPs had regarding CDP in Fiji. The findings of this study indicated that EDPs had very little information about CDPs and no proper details being provided to them during CDP treatment.

A couple of patients also commented that they had no information about CDP.

*"I don’t have much knowledge on this denture. Just how I clean and use that’s all I know about this teeth."* (P12)

Some participants stated that there was nothing explained to him by the DPs about complete denture rehabilitation when he went to the dental clinic to get his CDP made.

*" When I went to make my teeth they don’t tell me anything about the teeth. Because one of my aunty had false teeth like that so now I have to follow it and make mine."* (P13)

### Theme 2: non-compliance

This theme looked at the concerns the EDPs had raised while wearing their CDPs in Fiji. Occurrence of blisters, difficulty in closing the mouth and loose dentures were identified as the common causes for non-compliance which has compelled the patients not to wear the dentures.

A few patients found it very difficult to wear their CDP due to blisters.

*“Because I get blisters by wearing the lower dentures, when that happens i happen to take out the dentures and don’t wear it for some time and put it away. I am not able to eat anything when I start having blisters.”* (P14).

Some patients also mentioned that they could not close his mouth properly when he wore his CDP that was fabricated for him.

*"In the beginning, when I got my dentures, I could not wear it properly. I could not close my mouth fully while having the dentures on. This used to give me a lot of pain. It used to give me so much pain that used to start crying. It was that painful leading to headaches too."* (P20)

Few patients stated that their upper CDP is so loose that it comes out of his mouth.

*"I find my upper denture a bit slack. I feel its loose for me. Otherwise the lower denture is proper for me. When I sneeze or cough, the upper denture can come out of my mouth. This is one disturbance I face. Also in the beginning I used to have vomiting when I wear these dentures."* (P22)

### Theme 3: overwhelmed by illness

This theme highlighted how challenging it was for EDPs in Fiji to wear CDP due to undergoing numerous illness which hampered the appropriate utilization of dentures. Old age and poor oral structures together with additional health problems were featured under this theme.

A few patients who were really old and had flabby ridges had difficulties wearing their CDP.

*"Due to my old age and ridge resorptions I feel my dentures are getting loose which is causing problems in eating food now. I feel now the food is getting in between the denture and my bones where the denture sits and also end up with sore spots in the mouth."* (P21)

Some EDPs mentioned that they couldn’t go back to the dental clinic to get his CDP checked because of his additional health problems.

*"I was planning to come again to the clinic and complain about the denture but unfortunately I got admitted in the hospital for next 5-6 weeks. Therefore, I couldn’t go to clinic. I wanted to request them to either make my teeth again or fix the one side which is giving me pain."* (P9)

### Theme 4: reduction in QoL

This theme showed how the challenges faced by the EDPs in Fiji related to CDP brought about reduction in the QoL of these patients. Pain, discomfort and lower CDP were some of the highlights of this theme.

Numerous patients had stated that they initially faced a lot of pain when she had got herself a CDP.

*"Initially when my dentures were constructed, it was giving me a lot of pain. I went to New Zealand and got a denture glue for myself with the help of my sister. The name of the glue is Polident. I apply this glue and then wear my dentures in order to eat properly."* (P21)

Few patients mentioned that the CDP is quite uncomfortable for them to get adapted to.

*"When I first got this dentures, i felt so uncomfortable with it having a chunk of thick teeth being placed inside my mouth. I was facing personal disturbances from this as this has started to cause cuts and sore spots in my mouth. I was having headaches as well which were signs of sickness from this denture."* (P30)

Some patients also mentioned that the lower CDP are of no help to them.

*“The bottom denture is slack so it disturb me at home because denture is loose. it comes out of the mouth.”* (P25).

### Theme 5: financial barriers

This theme indicated the financial challenges faced by EDPs related to receiving CDP services in Fiji. High costs related to CDP treatment and social welfare assistance were two key features encountered under this theme.

EDPs highlighted on how expensive it was to get a CDP fabricated.

*“I had spent a lot of money on my other false teeth before this one.”* (P1).

The current cost of getting a denture is reasonable for many EDPs under the social welfare assistance scheme in Fiji.

*“My view on these dentures are good. Getting teeth is very expensive but I am a social welfare recipient so my dentures were made for free*." (P28)

### Theme 6: personal acceptance

This theme exhibited challenges EDPs had faced at a personal level during CDP service delivery in Fiji. Well fitted CDP was one of the focus area under this theme.

Majority patients were satisfied with their well-fitting CDP.

*“I really like this set of dentures that is made for me. The teeth sits well on my bones and I am able to do wear it properly and use every day."* (P2)

Some EDPs are also content with the CDP that is fabricated for them.

*"This teeth of mine is very good my dear. There was a small disturbance but then I went to the hospital and it was well taken care of and adjustments were done. Now it’s very good. I have no problems at all now."* (P4)

## Discussion

The aim of the study was to explore the challenges faced by the EDPs during CDP service delivery in Fiji. Some EDPs had reported that they had very less to no knowledge on the CDP they had received from their dentists on how to clean and maintain their CDP as well. Similar results were highlighted whereby it was found that 51.89, 77.5 and 82.9%, from the participants did not receive appropriate denture cleaning instructions from their respective dentists [26–28]. It had been found that generally, patients were not informed about CDP care and maintenance which led to lack of oral hygiene and poor perception of denture care in EDPs [29, 30]. One EDP had reported to be following her relative as she observed that at old age there is a need to have CDP in order to eat food properly. There has been no evidence in the literature found regarding this statement thus remains as source of family support in the community when getting treated for CDP.

This study found out that a certain number of EDPs did have CDP non-compliance issues related to poor design of complete dentures (broader issues, raised occlusal vertical dimension and poor retention) whereby they were unable to wear their denture properly. In a previous study, it had been highlighted that non-compliance of the patients is due to the denture teeth not set in the neutral zone which if taken into account tends to develop pain for EDPs [31]. The study also found out that EDPs concentration towards cleaning CDP was more prominent towards their old sets of CDP than the recent CDP that they had been wearing [26, 32]. The EDPs had also confirmed that they were made aware upon CDP insertion that there would be possibilities to experience discomfort initially but that would improve upon thorough wearing of dentures on a daily basis. Many patients had experienced difficulties in wearing CDP together with not being able to accept the minimal efficiency of CDP when compared to natural teeth which was replaced [33].

To add on, the study explored on how EDPs who were very ill faced difficulties in undergoing CDP treatment whereby being overwhelmed by illness significantly associates with it. In this study “overwhelmed by illness” related to how EDPs who were medically compromised and had other oral health problems underwent CDP treatment. One of the major factors affecting the care and maintenance of CDP for the EDPs were the other illness or diseases associated to the health whereby it was found that EDPs who were medically compromised or were being treated with medications that would produce oral side effects with further intraoral manifestations would experience more difficulties with their CDP than the non-medically compromised EDPs [34]. Dryness of mouth led to frequent falling of CDP. It had been highlighted that dryness of oral cavity is strongly correlated with tongue coating leading to development of bad odor [35–38].

Moreover, the study probed how the EDPs had poor QoL while receiving CDP treatment hence reduction in QoL rightfully links here. In this study, “reduction in QoL” related to challenges faced when wearing loose CDPs and how patient satisfaction relates to improved QoL. CDP have shown to provide a positive impact towards the QoL of the EDPs leading to patient satisfaction [39–41] as edentulism associates itself with oral health disabilities. It had been found that EDPs wearing CDP had reported better satisfaction and improved oral health than the partially EDPs having few natural teeth remaining in the dentition [42]. Loose CDP causes difficulty in eating which reduces the quality of life for the EDP whereby it had been stated that the fit, retention, comfort, stability and esthetic of a CDP will impose and overall impact on the quality of life of a EDP [43–45].

Furthermore, the study enlightened how EDPs had faced financial difficulties while seeking CDP treatment thus financial barriers to CDP treatment and care suitably correlates to it. In this study “financial barriers” related to how EDPs had to spend considerably to receive CDP treatment and with some EDPs where treatment was free of cost at the University Clinics while some EDPs had the CDP treatment cost subsidized to provide treatment affordability. EDPs had stated that they had spent a valuable portion of money in getting their CDP to assist them in their daily living where it had been found that EDPs have reported financial barriers to dental care than any other healthcare irrespective of age, income level or even the type of insurance an EDP had [46, 47]. Moreover, EDPs who visited FNU dental clinic did not spend any money in getting their CDP where these EDPs who had visited university dental clinics did not pay for the denture treatment cost [48]. In addition, EDPs had stated that the government had supported them financially in order to receive their CDP. Financial subsidies were provided by the government to assist the economically vulnerable EDPs to receive denture services at reduced cost whilst dental clinics with government subsidies utilized the fees that was paid by other EDPs to subsidize CDP treatment for those EDPs who cannot afford [49].

Lastly, the study explored how the EDPs had accepted the CDPs which were fabricated to improve mastication whereby personal acceptance appropriately relates to it. In this study “personal acceptance” related to how the EDPs appreciated having CDPs and how it improved the physical appearance and the confidence level in interacting with families and friends in public. The findings of this study showed that most EDPs personally accepted the CDP that was made for them to specifically fit them which states that physical appearance is equally vital for self-esteem as well as how people behave around an EDP [50]. In addition, EDPs’ acceptance towards CDP improves if they were well fabricated [51, 52]. On the other hand, EDPs also do not find the CDP accepting as the CDP was not made to fit the EDP appropriately. The adverse psychological reaction towards CDP by the EDPs have been identified where anxiety on rejection or avoidance would develop anger, sadness or even depression for the EDPs [50].

### Limitations

There are some limitations that must be acknowledged in this study. Firstly, the study included a handful of CDP patients. It would be ideal to future researchers in this area of study to include a larger sample size.. Secondly, due to global widespread of COVID-19 epidemic and accompanying restrictions, participants of the study were undertaken virtually via telephone call where verbal consent was received from the participating EDPs once the information sheet and consent form was fully read out the them. Thirdly, there was also inclusion of overlapping themes in the study and finally, due to the nature of the study participants, data collection was conducted based on participants’ availability for the respective day.

## Conclusion

The findings indicate on the challenges faced by EDPs during CDP service delivery in Fiji. The EDPs expressed a variety of challenges that was faced while seeking appropriate CDP services which were lack of information, non-compliance, overwhelmed by illness, reduction on QoL financial barriers to CDP treatment care and personal acceptance by the EDPs. The findings demonstrate the need to explore approaches that cultivate patients’ engagement during CDP service delivery by the Dental Professionals (DPs), as this will lead to effective oral health promotion in Fiji for CDP wearers. Additionally, DPs should be aware of the challenges faced by the EDPs when they sit on the dental chair and also to ensure that the dentures are designed in such a way to improve compliance, acceptance and quality of life for EDPs. Meanwhile, the role of family members and peers should be utilized to a reasonable extent to support EDPs engagement in achieving optimum CDP services in Fiji.

## Data Availability

The datasets generated and/or analysed during the current study are not publicly available due to privacy or ethical restrictions but are available from the corresponding author on reasonable request.

## Abbreviations

EDP: Edentulous Patients
CDP: Complete Denture Prostheses
DPCs: Dental Prosthetic Clinics
QoL: Quality of Life
DPs: Dental Professionals
WHO: World Health Organization
NCDs: Non-Communicable Diseases
COREQ: Consolidated Criteria for Reporting Qualitative Research
COVID-19: Coronavirus Disease 2019
FNU: Fiji National University
CWMH: Colonial War Memorial Hospital
HOS: Head of School
SDOH: School of Dentistry and Oral Health
PDO: Principal Dental Officer
TMJ: Temporomandibular Joint
IDI: In-Depth Interview
FID: Fijian of Indian Decent
IT: I-Taukei
PDW: Partially Denture Wearers
